# Predictive Role of Immune Profiling for Survival of Multiple Myeloma Patients

**DOI:** 10.3389/fimmu.2021.663748

**Published:** 2021-07-05

**Authors:** Liu Zhaoyun, Fu Rong

**Affiliations:** Department of Hematology, Tianjin Medical University General Hospital, Tianjin, China

**Keywords:** immunology, survival predication, immune profiling, multiple myeloma, prognosis

## Abstract

Despite new efficacy drugs and cell therapy have been used for multiple myeloma (MM) patients, some patients will relapse over time. We wonder the immune system play a vital role as well as MM cell during the development of disease. It is clear that the characteristic of myeloma cell is associated with the survival of MM patients. However, the link between the immune profiling and the prognosis of the disease is still not entirely clear. As more study focus on the role of immunity on multiple myeloma pathogenesis. There are plenty of study about the predictive role of immunity on the survival of multiple myeloma patients. Up to mow, the majority reviews published have focused on the immunotherapy and immune pathogenesis. It is indispensable to overlook the predictive role of immunity on multiple myeloma patients. Here, we give a review of vital previous works and recent progress related to the predictive role of immune profiling on multiple myeloma, such as absolute lymphocyte count, neutrophil-to-lymphocyte ratio, platelet-to-lymphocyte ratio, lymphocytes and cytokines.

## Introduction

Multiple myeloma is an incurable disease. With the rapidly increasing treatment choices available for MM patients, markedly strong responses or minimal residual disease (MRD) negativity can be achieved. This has resulted in the significant improvement of survival outcomes for patients. However, MM patients received autologous transplant to achieve low MRD will relapse finally. What is more, relapsed early (<18 mouth) are associated with infinite survival regardless of MM cytogenetic risk (1). That indicated the bone marrow micro environment including the immune system is pretty crucial in MM. The approaches necessary to perform a comprehensive evaluation to the prognosis of MM patients warrant further exploration. Standards now used, such as IPSS and R-IPSS, are related to myeloma cell characteristics. Several studies have revealed that the immune system is related to the proliferation of myeloma cells and is involved in the progression of the disease. However, a question arises on the aspects of the immune system that can help predict MM prognosis. Thus far, most reviews published on the immune profiling of myeloma have focused on immunotherapy and the role of immunity in the bone marrow micro environment. Few reviews have reported the value of immune profiling in the prognosis to be conducted for MM patients. In clinical practice, it is necessary to give a comprehensive evaluation to MM patients with the treatment of new drugs and autologous stem cell transplantation (ASCT). There is no doubt tumor burden is the most practical index in clinical. Several methods have been widely used to detect MRD such as flow cytometric MRD assay, Allele-Specific Oligonucleotides Real-Time Quantitative PCR and Next-Generation Sequencing. To make a comprehensive survival evaluation to MM patients we need identify some immune index duo to the irreplaceable immune role in MM. This review summarizes the immune index in the context of prognostic significance in MM patients (**Table 1**).

**Table 1:** Summary of immune profiling in the prediction of survival of patients with multiple myeloma.

	Index	Patients(n)	Threshold value	Time to collect sample			Prediction of survive			Sample		Reference
				New diagnosis	Induce therapy	Post-Autologous stem cell transplant	OS	PFS	Other	Period blood	Bone marrow		
**1**	ACL	537	>1.4x109/L *VS* <1.4x109/L	√			65 *vs* 26 months			√		(2)
**2**	ACL	38	>0.8x109/L *VS* <0.8x109/L		day 29		58.3 *vs* 42.5 months			√		(3)
**3**	ACL	769	≥1400 cells/µL *VS* <1400 cells/µL			day-0, day-15 and day-90	111, 90.7 and 84months versus 74, 70.5, and 65 months			√		(4)
**4**	ACL	59	≥1000/mm3 *VS* <1000/mm3			day-23	37.96 *vs*. 23.19 months	18.72 *vs*. 9.11 months		√		(5)
**5**	ALC	125	<1.4*10^9 *vs* ≥ 1.4*10^9	√			63.2 *vs* 58.5 months	2-year PFS: 90.2% *vs* 86.3%		√		(6)
**6**	NLR	119	<2 *VS* ≥2	√			5-year: 87.5% *vs* 42.4%		5-year(EFS): 88.4 *vs* 41.8%	√		(7)
**7**	NLR	309	≥2 *VS* <2	√				22.1 *vs* 43.4 months		√		(8)
**8**	NLR	161	>2.78 *VS* ≤2.78	4 weeks before/after				median survival: 37 months *vs* 66 months		√		(9)
**9**	NLR	559	>4 *VS* ≤4	√			43.2 *VS* 56.0 months	24.03 *VS* 37.46 months		√		(10)
**10**	NLR	273	≥2.25 *VS* <2.25	√			16.0 *VS* 44.2 months			√		(11)
**11**	NLR	52	≤1.72 *VS* >1.72	√			42.75 *VS* 26.14 months			√		(12)
**12**	NLR	76	<2.95 *VS* ≥2.95	√			4-year rates: 64.8% *VS* 30.9%		CR rate: 39.2% *VS* 20%	√		(13)
**13**	NLR	176	>1.51 *VS* <1.51		√		2-year rates: 72.2% *VS* 84.7%		CR rate: 7% *VS* 26.1%	√		(14)
**14**	NLR	315	≥2 *VS* <2	√			18 *VS* 29 months	12 *VS* 20 months		√		(15)
	PLR		≥119 *VS* <119				no statistical significance	no statistical significance				
**15**	LMR	285	≤4.2 *VS* >4.2	√			3-year: 64.2% *VS* 77.3%	3-year: 37.9% *VS* 68.1%		√		(16)
**16**	NLR	150	≥1.46 *VS* <1.46			day-100	37 *VS* 48 months	24 *VS* 36 months		√		(17)
	PLR		≥86 *VS* <86				37 *VS* 57 months	25 *VS* 38 months				
	MLR		≥0.27 *VS* <0.27				34 *VS* 51 months	25 *vs* 36 months				
**17**	CD57+cells	75	continuous variable(elevated)		√			superior			√	(18)
**18**	CD19+ B-cell	521	<125 *VS* ≥125 cells/ml	√			2.8 *VS* 4.0 years		event-free survival: 2.0 *vs* 2.7 years	√		(19)
**19**	CD19+ B-cell	101	highest *VS* middle *vs* lowest quartile			pre-transplant	2-year: 93% *VS* 90% *VS* 63%	2-year: 83% *VS* 59% *VS* 53%		√		(20)
**20**	Bregs	29	<10% *VS* ≥10%	√			20 months: nearly 80% *VS* 100%				√	(21)
**21**	sBCMA	184	<326.4ng/ml *VS* ≥326.4ng/ml	√			155 *VS* 96 months 139 *VS* 92 months	9.0 *VS* 3.6 months 7.0 *VS* 3.1 months		√		(22)
**22**	median Treg frequency	66	≥6.16% *VS* <6.16%	√			20 months *VS* median not reached			√		(23)
**23**	Treg cell	44	≥5% *VS* <5%	√					TTP: 13months *vs* median not reached	√		(24)
**24**	Treg levels of CD4+T cell subsets	53	>14.6 *VS* ≤14.6			before the first DLI	25.5 *VS* 67 months	4.2 months *VS* 12.4 months		√		(25)
**25**	NK cell activity	29	≤10 *vs* 11-20 *VS* >20		√		25-30 *VS* 60-65 *VS* 55-60 months			√		(26)
**26**	NK cell count	114	<100/uL *VS* 100 to 200/uL			a month		2.2 *VS* 11.6 months		√		(27)
**27**	γδT cell	101	highest *VS* lowest quartile			day-100	2-year: 89% *VS* 65%	2-year: 65% *VS* 45%		√		(20)
**28**	m-MDSC	100	high *VS* low			pre-transplant			3-year TTP: 34.2(median: 27.1) *vs* 52.9%(median: not reached)	√		(28)
**29**	sIL-2R	81	>6.049 *VS* ≤6.049	√				11 *VS* 24 months	ORR: 41.7% *VS* 60.0%	√		(29)
**30**	IL-6	42	<7pg/ml *VS* ≥7pg/ml	√					50% survival rate: 57.3 *VS* 2.7 months	√		(30)
**31**	FGF-2、VEGF	124	FGF-2 ≤ 950 and VEGF ≤ 19000pg/dl *VS* FGF-2>950 or VEGF>19000pg/dl *VS* FGF-2>950 and VEGF>19000pg/dl”	√			67 *vs* 55 *vs* 37 months	38 *VS* 24 *VS* 15 months		√		(31)
**32**	BAFF	52	>847.98 *VS* ≤ 847.98pg/ml	√				shorter(nearly 1400 days) *VS* longer(1600-1800 days)		√		(32)
	APRIL		>2.26 *VS* ≤ 2.26ng/ml					shorter(nearly 1600 days) *VS* longer(nearly 1800 days)				
**33**	IL-10	188	>169.96 *VS* ≤169.96pg/ml	√			3-year: 51.9% *VS* 93.6%	3-year: 13.3% *VS* 69.3%	ORR: 53.3% *VS* 79.2	√		(33)

## Cellular Immune Profiling to Predict MM Prognosis

### Absolute Lymphocyte Count (ALC)

ALC reflects the restoration of hematological parameters after autologous peripheral blood (PB) stem cell transplantation, and is an independent prognostic factor for clinical outcome. A retrospective study conducted in 537 newly diagnosed MM patients in the Mayo Clinic indicated that the survival of MM patients with an ALC > 1.4 × 10^9^/L was associated with better overall survival (OS) (65 *vs*. 26 months) (2). Another study revealed the same results, in which, after induction therapy day (D) 29, 38 MM patients with an ALC > 0.8 × 10^9^/L had better OS (58.3 *vs*. 42.5 months) (3). Patients with ALC ≥ 1400 cells/µL or <1400 cells/µL at post-autologous stem cell transplant at D0, D15, and D90 experienced a different OS (111, 90.7, and 84 months *vs*. 74, 70.5, and 65 months, respectively) (4). In D23 of post-autologous stem cell transplant, MM patients with ALC ≥ 1000/mm^3^ *vs*. < 1000/mm^3^ also showed a different OS (37.96 *vs*. 23.19 months) (5). However, ALC in 125 older MM patients treated at diagnosis with IMIDs and not eligible for autologous stem cell transplantation (ASCT) is unrelated to OS (6). ALC could reflect host immune function. The survival of MM cells mostly affected by their interaction with the immune micro environment. ALC can be a most reported index to predict the survival of MM patients. However, there some question need to be solved carefully. 1) what is the time we should detect ALC, new diagnosis point or post-autologous stem cell transplant at some day? 2). As we all known that the recovery of immunological cell after hematopoietic cell transplantation need almost 1 year (34),current data need extend the follow up time to further observe the survival predictive value of ALC in MM.

### Neutrophil-to-Lymphocyte Ratio (NLR)

The NLR has been reported as an adverse prognostic factor among cancer patients (35–37). Lately, NLR has been reported to possess prognostic value in patients with MM: high NLR is associated with the score of the ISS system in newly diagnosed MM patients. Furthermore, MM patients have shortened OS with high NLR (7–12, 14, 38).

Engin Kelkitli et al. (39) first reported that, in 151 MM patients, the NLR was significantly higher than that in healthy controls (2.79 ± 1.82 *vs*. 1.9 ± 0.61). However, because the patients included in these studies were not in full accord, we did not obtain an accurate and concrete NLR value. Romano et al. (8) showed that the median NLR in 309 newly diagnosed MM patients was 1.9 (range: 0.4–15.9). This was similar to another study that evaluated the median NLR in 131 MM patients and the value was estimated to be 1.93 (range: 0.10–36.23) (9). In a recent study, 559 MM patients were included. The NLRs of patients prior to therapy and 123 healthy controls were 2.096 ± 0.0629 and 1.771 ± 0.0747, respectively (10). As NLR data were obtained by estimating complete blood count (CBC), differences in NLR might result from the different numbers of MM patients considered in the various studies; however, the results indicated an increase in NLR in MM patients.

The NLR at diagnosis can help predict the survival of patients with MM. Several studies have revealed that a high NLR is associated with shorter OS or PFS. In several studies (7, 9, 11), multivariate analysis for OS showed that high NLR was an independent significant prognostic factor. Shi et al. (10) performed a multivariate analysis, which indicated that elevated NLR was a statistically independent predictor of PFS. Engin Kelkitli et al. revealed that the duration of OS and EFS in MM patients with NLR ≥ 2 was shorter than that in patients with NLR < 2 (5-year OS: 87.5% *vs*. 42.4%; 5-year EFS: 88.4% *vs*. 41.8%) (7). In a similar study, the 5-year PFS and OS were 18.2% and 36.4% in 309 newly diagnosed MM patients with NLR ≥ 2 compared to 25.5% and 66.6% in patients with NLR < 2 (8). In another report, 52 MM patients were enrolled, and patients with NLR ≤ 1.72 at diagnosis obtained superior OS rates than MM patients with NLR > 1.72 (42.75 *vs*. 26.14 months) (12). In a study involving 559 MM patients, Shi et al. reported that the median PFS and OS were significantly shorter among MM patients with NLR > 4 compared to the rest of the cohort (PFS: 24.03 *vs*. 37.46 months; OS: 43.2 *vs*. 56.0 months) (10).

As for the value of NLR in the treatment of MM patients, there have been a few studies conducted on the application of drugs and ASCT. The application of novel drugs, such as proteasome inhibitors and immunomodulatory agents, is a remarkable improvement in MM treatment. As a result, the OS of MM patients has shown recent improvements (2). Zhou, X. in 2018 (13) investigated 76 newly diagnosed MM patients to study the association between NLR and OS. In multivariate analysis, NLR was not an independent prognostic factor for poor survival of patients receiving bortezomib-based therapy; patients obtained lower 4-year OS rates with NLR > 2.95 compared to patients with NLR < 2.95 (30.9% *vs*. 64.8%). However, in a prior study involving 179 MM patients treated with the bortezomib-melphalan-prednisone (VMP) regimen, multivariate analysis revealed that high NLR was an independent poor prognostic factor (14). In addition to the OS, it also has an indication of the treatment response to bortezomib. The complete response rate (CRR) was significantly inferior when comparing high- and low-NLR groups (7% *vs*. 26.1%). Based on the results of univariate logistic regression analysis, as well as multivariate analysis, high NLR was identified as a poor prognostic factor of CRR to bortezomib (14). In the last decade, ASCT has been widely used in treatment of MM patients. An increased NLR indicates inferior survival of MM patients with ASCT (8, 17). Romano, A. et al. showed that the median PFS was significantly shorter for MM patients with NLR ≥ 2 compared to those with NLR < 2 (22.1 *vs*. 43.4 months) at diagnosis, when considering the clinical outcomes of ASCT (8). In 2018, Solmaz Medeni, S. (17) conducted a study involving MM patients who underwent ASCT. High NLR at the 100^th^ day post-transplantation is associated with inferior OS and PFS.

In summary, for newly diagnosed or post-ASCT MM patients, increased NLR may help predict inferior clinical outcome, and it may be used as a prognostic biomarker for the prediction of survival and treatment response owing to the low cost and rapidity of the test. However, the time to detect NLR and the cutoff value should be further standardized before it can be used in clinical practice.

### Platelet-to-Lymphocyte Ratio (PLR)

The predictive role of PLR in MM patients remains controversial. A previous study revealed that PLR could be used as an independent prognostic factor in several cancers. A retrospective study that enrolled 175 MM patients revealed that the median PLR was 127.69 (range: 0.46–1959.60). Furthermore, the best cutoff value of PLR for OS by ROC curve plot was 155.58. MM patients with a PLR> 155.59 also showed lower albumin levels and higher survival staging (9). A high PLR on the 100^th^ day post-transplantation indicated an inferior clinical outcome in 150 MM patients after ASCT (39). However, in another study that enrolled 315 newly diagnosed MM patients, there were no significant differences in OS or PFS, in contrast to PLR. In this study, NLR might be used as a superior index to predict survival than PLR. Based on the results of multivariate Cox analysis, it can be inferred that PLR is not a useful independent prognostic factor and it cannot be used to predict the OS and PFS of MM patients (15). All those results may because PLR takes both promote tumor status and anti-tumor immune status into consideration,which lead this index become not exactly since there need take platelet and lymphocyte into consideration at the same time.

### Monocyte-to-Lymphocyte Ratio (MLR)

MLR is another useful index for predicting the survival of individuals upon diagnosis (40). In a previous study that enrolled 285 MM patients (16) at a diagnosis phase, patients with MLR ≥ 0.24 had shorter OS and PFS than patients with MLR < 0.24. Additionally, multivariate analysis revealed that MLR ≥ 0.24 could be used as an independent predictor for OS and PFS. In another study that enrolled 150 MM patients after ASCT, at the 100^th^ day of a post-transplantation period, MLR ≥ 0.27 indicated an inferior clinical outcome (17).

### T Lymphocytes

Numerical and functional T cell abnormalities have been described in MM patients, which results in the occurrence of immune dysfunction, such as disrupted immune surveillance and immune escape, and can even lead to disease progression (41, 42). Abnormal T cell repertoire owing to an abnormal ratio between CD4^+^ and CD8^+^ T cells, a decrease in the number of CD4^+^ T cells, and an increase in the number of regulatory T cells (Tregs) have been reported in previous studies in MM patients (41, 43, 44). As for functional dysfunction, the abnormally high expression of immune checkpoints, such as programmed cell death ligand 1 (PD-L1), leads to the inhibition of CTL cells in MM (45).

The number of CD4^+^ and CD8^+^ T cells plays a role in predicting the survival of MM patients. Schmidmaier R. et al. used flow cytometry to detect PB lymphocytes and aphaeresis products (AP) in 41 MM patients. Increased number of CD4^+^ cells and increased ratio of CD4/CD8 are significantly correlated with prolonged EFS. However, a high proportion of HLA-DR positive lymphocytes is negatively associated with EFS and OS (46).

The amplified T cell clones improve the survival of MM patients, and T cell expansion is predominantly CD8^+^ (93%). The expansions are associated with a significant prolongation of PFS and OS. This finding was similar to that observed in the thalidomide arm amplification group (47). In a clinical trial, 75 patients with relapsed/refractory myeloma received thalidomide. Elevated vascular endothelial growth factor (VEGF) baseline values help predict excellent RR and PFS. The increased number of CD57^+^ cells indicates a better PFS (18). Another subgroup of T cells also play a role in predicting survival. A study of 85 autologous HPCT patients (including 11 with MM) revealed that the number of CD4^+^ T cells pre- transplantation was associated with PFS and OS in patients with hematologic malignancies. The number of pre- transplantation memory T cells (CD4^+^CD45RA^-^CD62L^-^) was associated with PFS (48).

However, there need more studies focus on immune checkpoints such as CTLA4, TIGIT, and VISTA, and their roles during the progression of myeloma. Those results can reveal the role of immune cell in MM patients survival prediction.

### B Lymphocytes

A previous study revealed that OS was 2.8 years for low counts of CD19^+^ B cells (<125 cells/mL), whereas the OS for the high-CD19^+^ B cell count group (> 125 cells/mL) was 4.0 years in newly diagnosed MM patients (19). Based on the data obtained for 101 consecutive MM patients, the increased total PB counts of CD19^+^ B cells at pre-AHSCT is significantly associated with improved 2-year PFS (83% *vs*. 53%) and OS (93% *vs*. 63%). The same result was observed in bone marrow samples of MRD-positive patients as determined by flow cytometry pre-AHSCT. However, there was no association observed between pre-AHSCT total PB CD19+ B cell count and PFS or OS for MRD-negative patients. This indicated that a high B cell count improved the therapy outcomes. Higher counts of sub populations of B cells, including naive and memory B cells, in PB are associated with a superior survival and improved values of 2-year PFS and OS (20). CD19^+^ B cell counts in the PB and bone marrow are significantly higher in long-term disease control MM patients than those in healthy donors or MGUS (49).

Not only B cells, but also the B cell subgroup are associated with OS in MM patients. A real word data from CHINA analysis bone marrow Bregs in 29 MM patients. Patients with < 10% (0-62.1%) Bregs (within CD19^+^B-cell compartment) had significantly worse OS (21) with levels of serum B cell maturation antigen (sBCMA) are associated with PFS and OS in MM patients; sBCMA > 326.4 ng/mL had inferior PFS and OS (22).

All the data indicated that B cell (including B cell sub population) may play a vital role in the development of MM. Since plasma cell come from B cell. More B cell population may inhibit the proliferation of malignant plasma cell directly or indirectly, another possible reason may the decrease of malignant plasma population make the population of B cell or normal plasma cell increased. It still need further study to reveal the underlying mechanism of how B cell regulate the proliferation of MM cell. But at least, our present data can exactly indicate that B cell can well predict the prognosis of MM patients.

### Regulatory T Cells (Tregs)

Tregs are a sub population of T cells that control autoimmune reactivity *in vivo* and can suppress immune responses by directly interacting with other immune cell types or by the secretion of immuno suppressive cytokines. It is divided into naturally regulated T cells (nTregs) and induced or adaptive regulatory T cells (aTregs or iTregs).

In MM patients, Tregs showed no significant association with major clinical and laboratory characteristics after a median follow-up of 33 months. From a functional perspective, Tregs exhibit potent inhibitory effects regardless of the disease status (50). In the CD4^+^ T cell subset, the balance between Th17 cells and immuno suppressive Tregs is an important factor in immune control of malignant tumors. There seems to be a clinical significance between Th17 and Tregs (51). A study conducted by Bryant and his colleagues found that long-term survival of MM patients (>10 years after diagnosis) was significantly associated with higher Th17/Treg ratio than patients who were subjected to follow-up for less than 10 years. Additionally, elevated levels of Tregs can help predict inferior OS and PFS in patients with MM (52). Giannopoulos et al. divided patients into two groups based on the median Treg frequency. The OS of patients with lower Treg frequency (< 6.16%) was shorter than in those with high Treg frequency (≥ 16%) (23). Similarly, Muthu Raja KR1 et al. found that patients with ≥ 5% Treg cells had a worse TTP. Univariate Cox regression model analysis showed that only PB Treg cells showed a prognostic role (24). To understand and ultimately utilize the aspects of the immune regulatory mechanism in transplanted MM patients, Franssen LE1 and colleagues retrospectively studied Tregs in 53 MM patients. The relationship between patients with the highest quartile of Treg levels (> 14.6% CD4^+^ T cell subsets) was significantly reduced in PFS and OS. The results of multivariate Cox regression analysis of OS indicate that Treg levels are an independent predictor of OS. High Treg levels exert a negative impact on OS (25). However, a study evaluated the expression in Tregs and Th17 cells of related markers such as FOXP3, CTLA4, and RORγt by performing quantitative real-time PCR. The expression of FOXP3 and CTLA4 in MM patients was found to be 6-fold and 30-fold higher, respectively, than that in the control group. There was no significant difference in the expression of RORγt and other genes related to Treg and Th17 cell subsets. Further univariate analysis showed that none of the CD4+ T cell-associated genes exerted an effect on the prognosis of patients (53). This might be related to the screening procedures and the number of samples included.

### NK Cells

Natural killer cells constitute an innate lymphocyte response to cancer and viral infections (54). Natural killer cell-mediated immune function is further deteriorated in advanced MM cases. Compared with MGUS and untreated MM, the number of PB NK cells in advanced disease is substantially reduced (55). NK cells remain functional in patients with MGUS and, during the progression of MM, NK cell function may notably alter and eventually inhibit the development of advanced disease. Additionally, NK cell activity is positively correlated with disease-free survival in patients with MM (26).

NK cell count is a predictive index for MM survival. MM patients, one month after allogeneic hematopoietic stem cell transplantation, with NK cell counts below 100 cells/mL show shorter PFS than patients with NK cell count ranging between 100 and 200 or with counts above 200 cells/mL (2.2 *vs*. 11.6 months) (27). NK cells are significantly more abundant in PB in MM patients with long-term disease than those in healthy donors or MGUS (49).

However, For the function of NK cell,NK cell-activating receptors such as natural killer group 2D (NKG2D), NKp30, and NKp44 have no evident prognostic prediction value.

### γδ T Cells

Gamma delta (γδ) T cells, which are innate immune cells, play an important role in anti-tumor immune surveillance (56). However, there are no significant differences in PB or BM γδ-T cell counts between MGUS and MM patients compared to those in healthy controls (49). Another study enrolled 101 MM patients and found that higher γδT cell, and CD4 ^+^ central memory (CM) cell counts after AHSCT approximately 100 days were related to a superior 2-year OS (20).

### Myeloid-Derived Suppressor Cells (MDSCs)

MDSCs are an important cell type described as a heterogeneous subset of immature myeloid cells (57). These cells are an important part of the MM micro environment and modulate MM cell survival and immune escape. For G-MDSCs, Giallongo et al., Brown et al., and Indu et al. evaluated their abundance in the PB or BM of MM patients with newly diagnosed or relapsed cases, and found that the abundance was significantly higher when compared to patients with MM in remission, MGUS, and healthy subjects (57–60). Binsfeld et al. obtained similar results in murine MM models (61). In other studies conducted, M-MDSCs (CD14^+^ monocytic) were regarded as more important than G-MDSCs (CD15^+^ granulocytic) (62). Wang et al. and Anderson et al. reported that the abundance of M-MDSCs in PB or BM in newly diagnosed and relapsed MM patients were significantly increased compared with those in MM patients in remission as well as those in healthy donors (63). More importantly, they revealed that M-MDSC count was significantly related to disease activity and tumor progression (64). Lee et al. also revealed that the increase in peripheral M-MDSCs was associated with failure to achieve a response of VGPR or greater, suggesting poorer efficacy of the therapy (65). Min et al. and LE et al. demonstrated that pre-ASCT M-MDSC burden was correlated with a higher ISS stage and a lower TTP after ASCT (25, 28).

### Macrophages, BMSCs, OC Cells, and OB Cells

Macrophages contribute to the development of MM-associated neovascularization through both the paracrine secretion of angiogenic factors (angiogenic pathway) and a vasculogenic pathway, and may therefore represent a significant target for conducting anti-neovessel treatments in MM (54). MM cells can influence the polarization of macrophages by increasing the expression of M2-related scavenger receptors. Additionally, macrophages that are modulated by MM cells can inhibit the proliferation of T cells and the production of IFNγ  (66).

BMSCs play a vital role in the development of MM patients. It has been revealed that the proliferative capacity of MM patient-derived MSCs is lower than that in healthy donors, accompanied by a decreased expression of MSC-related receptors (67). Other studies revealed that cross-talk established between BMSCs and MM cells can support the proliferation of myeloma cells by the secretion of IL-6, cell growth factor, and other factors (68, 69). For important fact, BMSC can down regulate immune function, such as BMSC can negatively regulate the CTL cell by PD-1/PDL-1 pathway in our previous research (70).

OCs increase the expression of immune checkpoints such as CD200 (CD200R), Galectin-9 (Tim-3), and PD-L1 (PD-1) during osteoclastogenesis, which can inhibit the activity of T cells by the immune checkpoint (71). Furthermore, OCs can secrete Galectin-9 and APRIL. Galectin-9 induces the apoptosis of T cells and leads to an increase in MM cells, and APRIL can induce the expression of PD-L1 in MM cells and aid their immune escape (72, 73).

It has been reported that OBs are related to the inhibition of MM immunity (74). Myeloma cells inhibit the differentiation and maturity of OBs (75), and the production of cytokines, chemokines, and inflammatory factors in the bone marrow micro environment is involved in the regulation of both cells (76).

Although there lot of evidence indicated that those cell involved in the regulation of immune function in MM patients. The roles in predicting prognosis of MM patients remain unknown owing to insufficient study and a long time follow up clinical data. Further long-term follow-up studies will provide evidence to support the utilization of these markers in MM clinical prognosis prediction.

## Non-Cellular Components

### Immune Cell Released Cytokines

Cytokines can secrete by immune cell and other type cell such as tumor cell, Endothelial cell and even smooth muscle. It can be divided into inflammatory (such as IL-1 and IL-6) and anti-inflammatory (such as IL-1Rα, IL-4, and IL-10) types based on their effects on disease progression (77). Different cytokines play various roles in patients with MM. Here we focus on some major cytokines secreted by immune cell in MM.

#### IL-1

Few studies have revealed the effect of IL-1 in the progression of MM (78). Downregulation of IL-1 expression leads to lower activity of IL-6 (79). A study conducted by John A. Lust et al., which enrolled 47 high-risk MM patients, showed that treatment with an inhibitor of IL-1 (anakinra) resulted in superior PFS (37.2 months) and OS (9.5 years) (80).In fact, it has been reported the IL-1 levels was a process reason for MGUS to SMM to active MM (78). Increased IL-1 may lead chronic inflammation which will lead emerge of DNA damage and induced the mutations DNA of tumor cell (81).

#### sIL-2R

sIL-2R levels in MM patients are notably increased compared with healthy donors (8.51 ng/mL *vs*. 0.56 ng/mL) in a study that enrolled 81 newly diagnosed MM patients. Additionally, ORR is significantly higher (60.0% *vs*. 41.7%) according to the best cutoff value of sIL-2R (6.049 ng/mL). Multivariate survival analysis indicates that sIL-2R levels are an independent prognostic factor for PFS. Furthermore, based on subgroup analysis results, high levels of sIL-2R are associated with poor PFS in MM patients (29). IL-2 can not only stimulate NK and T cell growth and enhance cytolytic action strongly, but also sensitizes T cells to activation-induced cell death and is required for Treg cells to reduce persistent immune responses (82). Since sIL-2R can bind IL-2 to decrease the immune effect stimulated by IL-2.

#### BAFF

P. J. Hengeveld et al. have reported that BAFF can be used as a biomarker for myeloma burden and for the estimation of the progression of disease (83). Patients with myeloma and higher concentrations of BAFF show worse PFS (32), mostly as BAFF promote the survival of both B cell (immature, naive and activated B cells) and MM cell by active the NF-B pathway (84). High level of BAFF concentration can lead the proliferation of MM cell.

#### IL-10

IL-10 is produced by several cells such as monocytes, NK, and T cells, and exerts anti-inflammatory effects (85). The concentration of IL-10 in high-risk MM patients is high (86). Multivariate analysis indicates that MM patients with high levels of IL-10 at diagnosis have an inferior PFS and OS (33). For mechanism IL-10 play a vital role in MM that IL-10 induces plasma cell proliferation and negatively regulate the antitumor host immune response (87, 88).

#### IL-17

Activated Th17 cells secrete most of the IL-17, although NK cells, CD8+ T cells, and neutrophils also generate variable quantities of IL-17. IL-17 induces the production of granulocyte colony-stimulating factor (G-CSF) and chemokines such as CXCL1 and CXCL2 and is a cytokine that acts as an inflammation mediator. During infection, IL-17 is needed to eliminate extracellular bacteria and fungus by inducing antimicrobial peptides such as defensin (88).

Lemancewicz et al. showed that IL-17A and IL-17E serum levels were significantly higher in all MM patients and also in patients with advanced stage compared with healthy subjects. They found the correlation between serum levels of IL-17A in MM patients and percentage of plasma cells. They also showed that if serum levels of IL-17E were higher in MM patients, the percentage of plasma cells and beta-2-microglobulin levels were lower (89). Alexandrakis et al. suggest that the elevated levels of IL-17 in BM and PB might be correlated with stage II and stage III MM. Another important finding of the present study was that the levels of IL-17 in BM and PB were significantly increased with the progression of MM (90).We have few cohort study data to demonstrated the level of IL-17 directly associated the survival of MM patients.

### Other Non-Cellular Components

Complex class I-related chain molecule A (MICA) is a ligand for NKG2D. A previous study revealed that the expression of MICA in plasma cells was related to the progression of MGUS to MM, and owing to a high expression of MICA in MGUS plasma, the immune response of NKG2D+ lymphocytes, such as NK cells, γδ-T-cells, and CTL cells (91, 92), could be induced.

Some tumor cell associated cytokines such as cytokines and angiogenic factors (CAFs)which can support the proliferation of tumor cell.FGF-2, HGF, VEGF, and PDGF-β plasma levels at diagnosis are indicative of more profound response since lower angiogenesis to MM cell. Furthermore, MM patients with low levels of FGF-2, VEGF showed superior PFS (81).

MM cell can also secrete cytokines.IL-6 plays a vital role in the proliferation of MM cells (79). It has been reported that high levels of IL-6 are related to disease progression. MM patients with high IL-6 levels (> 7 pg/mL) show inferior survival compared to patients with low levels (<7 pg/mL) (2.7 *vs*. 53.7 months) (80). Since IL-6/STAT3 signaling can promotes the creation of angiogenesis *via* enhancement of VEGF, the stimulation by IL-6/STAT3 signaling can also active MM proliferation related pathway like Ras, Akt and MAPK (93).

Other non-cellular components such as complement (94) and adiponectin (95) have been reported to be correlated with the development of MM. However, there are no data supporting its prognostic value.

## Immune Profiling and MRD

MRD detection plays a vital role in predicting the prognosis of multiple myeloma patients. Whether immunophenotype will affect the prognosis in MRD-negative MM patients or improve the transition from MRD-positive to MRD-negative remains to be determined. Few clinical studies have addressed this issue. Pre- AHSCT total PB CD19^+^ B cell count is associated with PFS and OS in MRD- positive MM patients (20). Another study finds an increased mature B lymphocytes will help MRD- positive MM patients experience prolonged survival (96) what is more, a similar result that the distribution of B-cell precursors were increased in both MRD- negative and positive MM patients reaching long-term disease control (49). For mechanism consideration, B cell is the precursor cell of plasma cell, more normal B cells (including B subgroup)may inhabit the proliferation of abnormal plasma cell. For this reason MM patients can achieve a long survival and get better clinical outcomes. However, is there any other type immune cell related to the MRD status and what is underlying mechanism? Further prospective studies are warranted to better understand the association between immunophenotype/IP and MRD status.

## Conclusions

**Figure 1:**
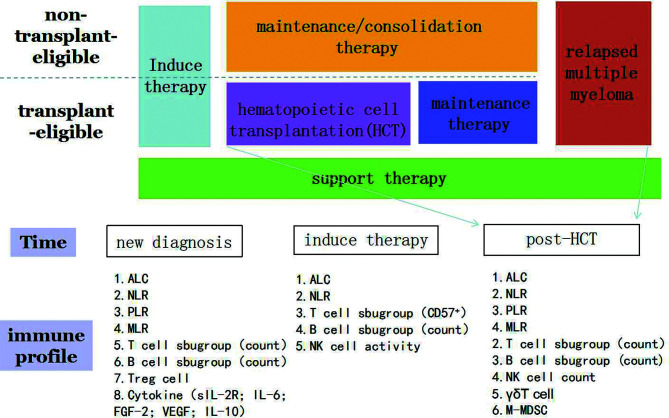
Immune profiling in the prediction of survival of patients with multiple myeloma. ALC, absolute lymphocyte count; NLR, neutrophil-to-lymphocyte ratio; PLR, platelet-to-lymphocyte ratio; MLR, monocyte-to-lymphocyte ratio.

With the rapid development and availability of new treatment choices for MM, MM patients will have the opportunity to achieve appreciable treatment responses. The Blood and Marrow Transplant Clinical Trials Network Myeloma Intergroup Workshop (BMT CTN Myeloma Intergroup Workshop) has indicated that MRD detection and immune monitoring are important for MM patients (97–99). Based on the discussion put forth by our review, we know that certain immune profiling such as ACL and B cell absolute counts can be used for the prediction of prognosis of real-world myeloma patients. Besides, some cytokines also play a vital role such as IL-1,IL-2 and so on (**Figure 1**). It is easy to be understand that immune cell can secrete cytokines to impact the survival of MM patients. Some cytokines can also regulate the function of immune cell. We can easily find a clue that immune profiling play a crucial role in the survival of MM patients. However, the following aspects should be considered and warrant further studies: a) most current immune indices focus on the number of immune cells to predict prognosis of MM patients, and not on the expression of function regulators (immune check points) such as PD-1, Tim-3, and TIGIT; b) data are mostly derived from studies conducted using peripheral blood, and not the bone marrow; c) the immune state, pre- AHSCT or post- AHSCT, has not well been evaluated; and d)As cytokines can regulate the function and proliferation of immune cell, on the other hand, immune cells can also secrete more cytokines, it still need to know the underling mechanism and mutual effect between cytokines and immune cell.

In future works, some advanced new technologies for multidimensional measurement will give more possibility to analyze the immune situation in MM patients. The full use of single cell RNA sequencing, genomic, immunophenotyping to evaluate the state of immune cells and proteins which might help to build an “immunogram” to evaluate immune status(including immune cell and cytokines) and even know the cancer-immune interactions in individual patients which may give a prediction of respond to immunotherapeutic strategies before clinical therapy and even play a vital role to predict the survival of MM patients. Only full considering these aspects can immune evaluation be performed to successfully predict survival in MM patients.

## Author Contributions

LZ: perception and drafting of the article, final approval of the version to be published. FR: participation in the whole work, revise the manuscript. All authors contributed to the article and approved the submitted version.

## Funding

This work was supported by the National Natural Science Foundation of China Youth Project (grant no. 81900131), the Tianjin Municipal Natural Science Foundation (grant no. 18JCQNJC80400), the Tianjin Education Commission Research Project (grant no. 2018KJ043), the Tianjin Education Commission Research Project (grant no. 2018KJ045), and the Tianjin Science and Technology Planning Project (no. 20YFZCSY00060).

## Conflict of Interest

The authors declare that the research was conducted in the absence of any commercial or financial relationships that could be construed as a potential conflict of interest.
